# A Proto-Architecture for Innate Directionally Selective Visual Maps

**DOI:** 10.1371/journal.pone.0102908

**Published:** 2014-07-23

**Authors:** Samantha V. Adams, Chris M. Harris

**Affiliations:** Centre for Robotics and Neural Systems, School of Computing and Mathematics, University of Plymouth, Plymouth, United Kingdom; University of Muenster, Germany

## Abstract

Self-organizing artificial neural networks are a popular tool for studying visual system development, in particular the cortical feature maps present in real systems that represent properties such as ocular dominance (OD), orientation-selectivity (OR) and direction selectivity (DS). They are also potentially useful in artificial systems, for example robotics, where the ability to extract and learn features from the environment in an unsupervised way is important. In this computational study we explore a DS map that is already latent in a simple artificial network. This latent selectivity arises purely from the cortical architecture without any explicit coding for DS and prior to any self-organising process facilitated by spontaneous activity or training. We find DS maps with local patchy regions that exhibit features similar to maps derived experimentally and from previous modeling studies. We explore the consequences of changes to the afferent and lateral connectivity to establish the key features of this proto-architecture that support DS.

## Introduction

The exploration of natural biological development and artificial developing systems are mutually informative. The self-organizing and high-dimensional open-ended learning ability of human development has been inspirational for artificial learning systems, and has led to the emergent area of developmental/epigenetic robotics (for a review see [1]). The concept of biological development has also been hugely influential in the field of evolutionary robotics spawning the Artificial Embryogeny sub-field [2]. Conversely, experiments in artificial networks and computational modeling have provided insight into how developmental processes could proceed. In particular, the ability of unsupervised self-organizing artificial neural networks to extract features from the natural environment has been highly influential, notably in the area of visual cortical maps [3]–[14]. Although exposure to the environment seems a compelling and sufficient condition for feature extraction, there is increasing evidence that it is not a complete explanation for natural development. There are now many examples in visual development of precocious abilities to extract visual features before visual experience has begun (for examples see the review in [15]). This raises the issue that the biological neonate may come equipped with a pre-conditioned “proto-“ architecture tuned to make the best use of its upcoming visual life.

Numerous studies have shown that many features of visual maps such as retinotopy (point-to-point topographic connections between the Lateral Geniculate Nucleus and V1), ocular dominance columns (OD) and orientation-selectivity (OR) circuits are already present at eye-opening (EO) and undergo further refinement with visual experience (for specific examples see [16], [17] and for a recent review see [15]). This early organization is thought to arise from two processes. Initially, molecular signals control the development of relatively coarse retinotopic mappings between and within the retina, superior colliculus, lateral geniculate nucleus (LGN), and primary visual cortex (V1). Later, spontaneous activity in the form of a series of retinal waves refines this nascent connectivity: see [18] for a comparison of the two mechanisms. Some previous computational studies have modeled pre-EO activity-dependent learning [11], [14], [19]–[21].

The development of maps for sensitivity to direction of motion (directional sensitivity, DS) is less clear with strong species differences. In the mouse, DS is present at EO and is not delayed by dark-rearing which implies it is independent of experience [22]. Retinal ganglion cells (RGCs) in the mouse retina exhibit directional selectivity [23]. Mouse cortical DS maps are not organized in cortical columns as is seen in other species, such as cat or ferret but individual cortical neurons do have clear preferences. It is not fully understood how retinal and cortical DS interact pre-EO in the mouse. The ferret, like other carnivores, is not known to have retinal DS and it is generally thought that dark-rearing delays DS development. In [24] it was reported that despite cortical DS being absent at EO, there was a very brief and early critical period with rapid development of DS within 2–3 weeks of EO. They found that dark rearing from EO over the critical period irreversibly disrupted the development of DS, but if normal visual input was restored within the critical period DS could subsequently develop. More recently, the work of [25] reported the existence of a weak cortical DS sensitivity and a neighbourhood bias at EO in ferret. However – as found by [24] - learning occurs rapidly after EO and it is possible that testing for DS may inadvertently have a training effect. Nevertheless, using a rapid sampling procedure the more recent work of [26] has confirmed the weak DS at EO. Both studies have shown that this initial DS facilitates later training for congruent stimulation. The origins of this weak DS and bias at EO are unknown: intrinsic cortical circuitry, retinal waves, spontaneous activity in the LGN and cortex and DS RGCs have all been mentioned.

Most previous modelling studies looking at the development of DS ([4], [6], [11], [20]) have not mentioned that pre-existing selectivity was present - some kind of activity-driven process has always been included as a requirement to produce a cortical DS map. Only [27] has attempted to explain initial DS without recourse to spontaneous neural activity or any kind of training process representing visual experience. They looked at how OR and DS could arise solely from an initial pattern of intra-cortical connections in an attempt to explain the apparent activity-independence of some maps that has been seen experimentally. Their results showed that localised ‘cortical’ patches responded to moving gratings in different directions and they produced DS maps similar to those seen experimentally. They stated that the OR/DS latent in the map architecture results from the cortical dynamics induced by ‘mexican hat’ connectivity (short range excitatory and long range inhibitory connections) and inhomogeneity in the lateral weights.

In this paper we describe a neural network similar to that of [27] but implemented with spiking neurons. Spiking Neural Networks (SNNs) use a neuron model which computes with pulses or spikes as real neurons do and are becoming increasingly popular due to their ability to model a range of biological phenomena. In particular the possibility to study the role of spike timing as there is now evidence that spike timing is important in behavioural contexts ([28]–[31]). Another incentive to use SNNs for the creation of bioinspired artificial systems is the increasing availability of neuromorphic devices which provide a means to implement large spiking networks directly in efficient low-power hardware.

In our work we concentrate mainly on how the structure of the network supports Directional Selectivity (DS) and apart from a different neuron model there are other key differences to the model of [27]: we have included distance-dependent delays on the intra-cortical connections and the contribution to neuron activity from the lateral connections is not emphasised over afferent input (as was the case in [27]) because we wanted to assess the relative contribution of afferent and lateral connection structure to DS. Our experimental results firstly confirm the results of [27] by showing that this network forms a proto-architecture for DS which manifests on presentation of moving input without the need for any activity-dependent learning and also that OR is present in the network. Additionally we show that both the form of the afferent connectivity from an LGN-like input layer and cortical lateral inhibition play a role in innate DS, with the cortical inhibition being the more important. Finally we discuss the relevance of these findings in relation to both biological and artificial systems.

## Methods

### The Visual Map Architecture

**Figure 1:**
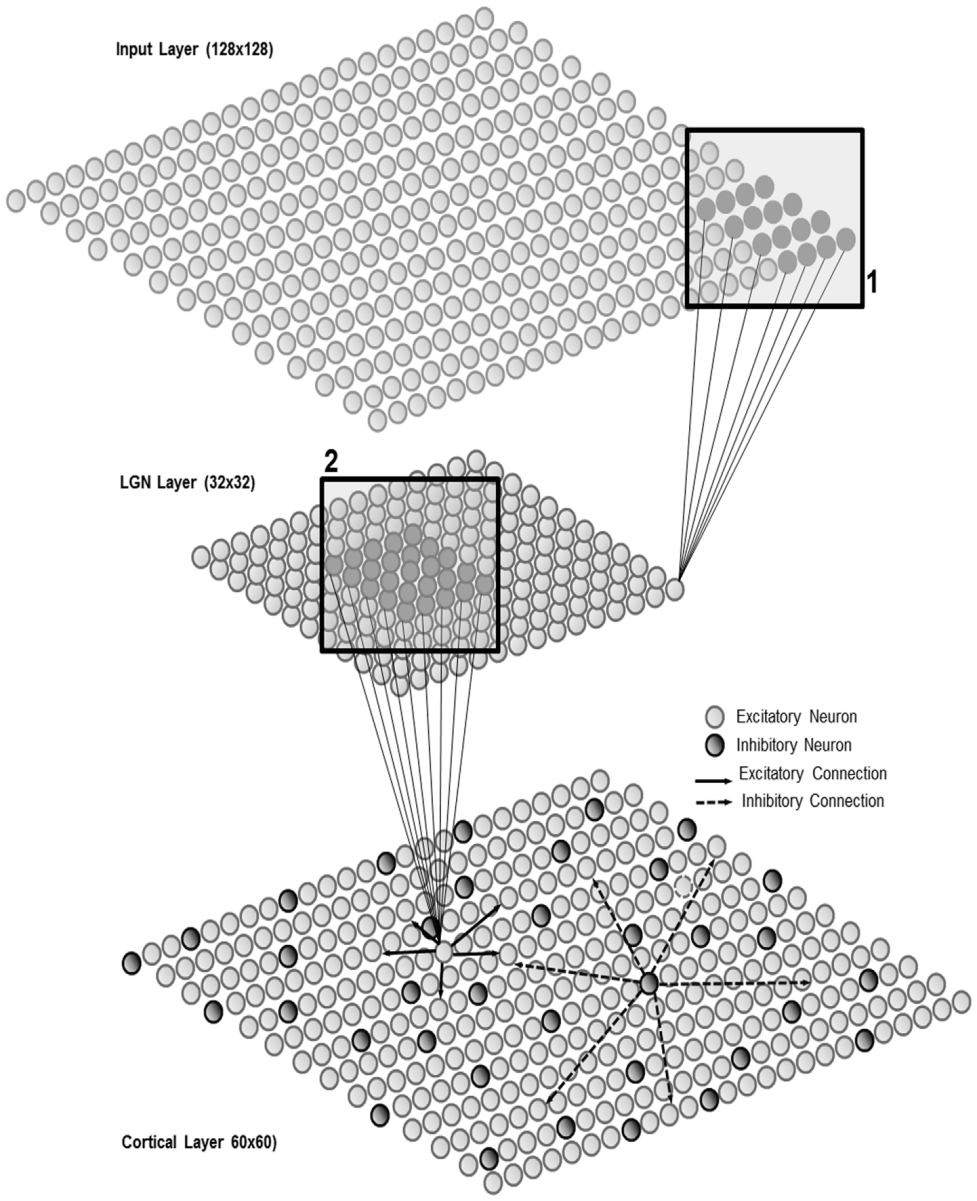
Visual Map Architecture. The network consists of 3 2D layers of neurons. The Input (top) layer receives spikes directly from the DVS 128 Silicon Retina camera at its resolution of 128×128 pixels. To achieve downsampling to 32×32 resolution, the Input layer neurons are connected to LGN layer neurons (middle layer) at a ratio of 16∶1 using a 4×4, non-overlapping connection field (box labelled 1.). The LGN layer neurons are in turn connected to the Cortical map layer (bottom) with overlapping 5×5 connection fields (box labelled 2.). All connections between layers are feedforward and excitatory. The Cortical layer consists of both excitatory and inhibitory neurons in a ratio 5∶1 and these neurons are connected sparsely to each other with excitatory neurons making short-range connections to nearby neighbours and inhibitory neurons making connections to neurons further away.

The network structure has been inspired by the Kohonen Self-Organizing Map (SOM) architecture [32], similar to that used in [33] but with some modifications to accommodate input from a DVS 128 ‘silicon retina’ camera. Figure 1 shows a diagram of the network architecture. The first layer consists of 128×128 neurons (referred to henceforth as the Input layer) and its purpose is merely to relay input spike data into the network at the same resolution as the DVS camera. The second layer consists of 32×32 neurons (referred to henceforth as the LGN layer) and its purpose is to achieve a down-sampling of the raw input data. The output or ‘map’ layer (referred to as the Cortical layer) consists of 60×60 neurons of which 20% are randomly assigned as inhibitory and 80% as excitatory as these are believed to be the proportions of inhibitory to excitatory neurons in real cortex [34]. Feed-forward excitatory connections exist between all three layers. The layers are not fully connected but instead there are connection fields (CFs) where neurons in a layer are connected to a subset of neurons in the previous layer. The Input layer is connected to the LGN layer with excitatory connections with fixed weights of value 1.0. These connections are set up such that a 4×4 connection field (CF) from the Input layer is connected topologically to 1 neuron in the LGN layer. These CFs are not overlapping, thus each neuron in the LGN layer averages the activity from 16 pixels in the Input layer. The box marked 1. in Figure 1 shows an example of one such set of connections. The neuron time constant and refractory period for the LGN layer neurons are set to ensure that there is no multiple firing in the LGN layer: i.e. any activity in the 4×4 group of input neurons results in 1 spike in the LGN Layer neuron.

Similarly, the LGN layer is not fully connected to the Cortical layer, but, in keeping with the approach of previous works modelling the visual system each cortical neuron only ‘sees’ neurons from the LGN layer within its connection field. The CFs from each cortical neuron overlap: see the box marked 2 in Figure 1 for an example. In our experiments, a 5×5 square connection field has been used as the ‘standard’ case. Afferent connection weights are set to an initial random value between 0.4 and 0.5. The Cortical layer is recurrently connected: there are sparse lateral connections and these follow a ‘mexican hat’ profile of short-range excitation and long-range inhibition. Excitatory and inhibitory connectivity is determined by probability functions based upon distance between the two neurons as given in equations 1 and 2.
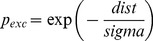
(1)

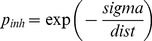
(2)


Where:


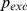
 is the excitatory connection probability (between 0 and 1.0)


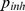
 is the inhibitory connection probability (between 0 and 1.0)

*dist* is the Euclidean distance between the neurons

*sigma* is the spread

**Figure 2:**
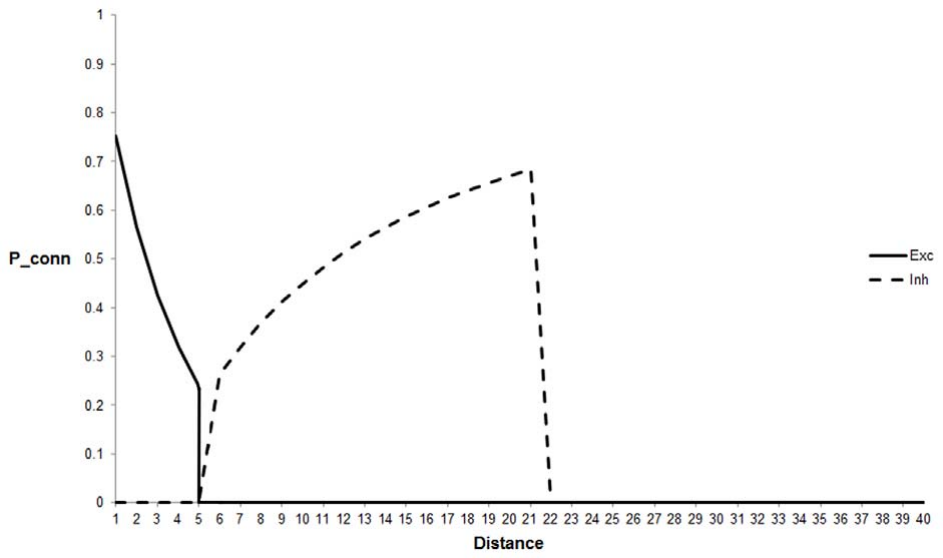
Connection probability profiles for the cortical layer. This figure shows the relationship between the cortico-cortical excitatory and inhibitory connection profiles. The curves represent the probability of connection (ordinate) by distance (abscissa). At distances less than 5 excitatory connection probabilities are high but at greater distances they fall to zero (solid line). At distances less than 5 the probability of inhibitory connections is zero, between 5 and 21 the probability increases and then falls to zero (dashed line).

For our ‘standard’ case, Cortical excitatory connectivity uses a sigma of 3.5 which gives a significant chance of connection at distances up to 5 units. At distances greater than this the probability is forced to zero. For Cortical inhibitory connectivity a sigma of 8.0 is used and at distances less than 5 units and greater than 21 units the probability is forced to zero. Figure 2 shows the profile of connection probabilities generated by this method.

Lateral connection weights are set to an initial random value between 0.3 and 0.4. Lateral connections also incorporate delays which are calculated according to the distance between the two neurons with added Gaussian noise. Refer to Table 1 for a summary of all the network parameters and their initial values.

**Table 1:** Summary of network architecture parameters.

Parameter	Value
N_in_, number of neurons in Input layer	16384 (128×128)
N_l_, number of neurons in LGN layer	1024 (32×32)
N_c_, number of neurons in cortical layer	3600 (60×60)
W_aff_, afferent synaptic weights	Randomly initialised between 0.4 and 0.5
W_lat_, lateral synaptic weights	Randomly initialised between 0.3 and 0.4 (exc) and -0.3 and -0.4 (inh)
Exc_p_conn_, connection probability for lateral excitatory connections	Calculated as *exp(-dist/sigma)* where dist is the Euclidean distance between the neurons and sigma is 3.5
Inh_p_conn_, connection probability for lateral inhibitory connections	Calculated as *exp(-sigma/dist)* where dist is the Euclidean distance between the neurons and sigma is 8.0

The network was implemented using the Brian spiking neural simulator [35].

### Neuron Models

LGN layer neurons are represented by a simple Leaky Integrate and Fire (LIF) model (equation 3).
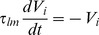
(3)


Where:


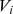
 is the membrane voltage of the LGN neuron


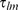
 is the LGN neuron membrane time constant

This is essentially a simple decaying voltage with spikes injected from connected neurons in the Input layer. When a presynaptic (Input) neuron fires the membrane voltage, 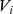 of the postsynaptic (LGN) target neuron is increased as shown in equation 4.
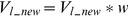
(4)


Where:


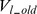
 is the original membrane voltage


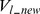
 is the updated membrane voltage

*w* is the synaptic weight

Synaptic weights are fixed at 1.0 for all connections. The membrane time constant 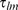 is set at 10 ms and the refractory period for LGN neurons is also 10 ms. This setup ensures that the first firing of any Input neuron in the 4×4 group connected to the LGN neuron will cause the LGN neuron to fire but immediate firing of other Input neurons in the group within the refractory period will not cause additional spikes in the LGN neuron.

For the Cortical layer neurons a simple Leaky Integrate and Fire (LIF) model based upon the well-known Vogels and Abbott CUBA (CUrrent BAsed) model [36] is used and described by equation 5.
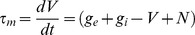
(5)


Where:

*V* is the membrane voltage


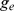
 is the contribution from excitatory synapses


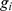
 is the contribution from inhibitory synapses

*N* is exponential noise


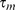
 is the membrane time constant

The neuron receives input from both excitatory (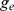) and inhibitory (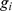) synapses which are represented by the fast AMPA (α-amino-3-hydroxy-5-methyl-4-isoxazolepropionic acid) receptor model which assumes that the action potential generated by the presynaptic neuron is instantaneous and decays exponentially over time in between further action potentials [37]. Following the method used in [9] a positive noise term *N* is added to the model to simulate background noise present in the cortex. This noise term is exponentially distributed and generated by equation 6.
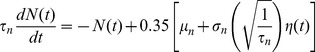
(6)


Where:

*N(t)* is the noise at time t


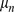
 is the mean of the noise


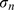
 is the standard deviation of the noise


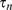
 is the time constant


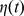
 is Gaussian white noise.

Synaptic dynamics are represented by equation 7.
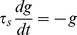
(7)


Where:


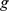
 is the effective conductance for an excitatory or inhibitory synapse


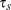
 is the synaptic time constant

When a presynaptic neuron fires the effective conductance (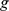) for excitatory and inhibitory synapses is updated as shown in equation 8.
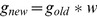
(8)


Where:


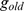
 is the original effective synaptic conductance


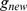
 is the updated effective synaptic conductance

*w* is the synaptic weight

Refer to Table 2 for a summary of the neuron model parameters and their initialised values.

**Table 2:** Summary of neuron model parameters.

Parameter	Value
V_reset_, reset voltage (LGN and cortical)	0 mV
V_ThreshL_, neuron threshold (LGN)	0 mV
V_ThreshC_, neuron threshold (cortical)	Randomly initialised as 1.0 mV plus noise normally distributed between 0 and 0.3 mV
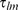 , membrane time constant (LGN)	10 ms
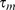 , membrane time constant (cortical)	5 ms
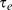 , excitatory synaptic time constant	5 ms
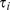 , inhibitory synaptic time constant	5 ms
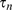 , noise time constant	5 ms
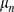 , noise mean	0.7
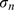 , noise stadard deviation	0.5
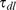 ,delay on lateral synapses	Set as distance between pre and postsynaptic neuron plus noise added by a Gaussian with mean 0 and standard deviation 0.5
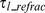 , neuron refractory period (LGN)	10 ms
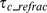 , neuron refractory period (cortical)	5 ms

### Input Patterns

In the main, previous works have used artificially generated moving bars or gratings as input to create directionally selective feature maps (for example, [6], [9], [11], [12]). A novel feature of the visual system in the current work is that the input is generated directly as spikes by a DVS 128 silicon retina camera [38], [39]. This device has been developed within the domain of neuromorphic engineering and has only very recently begun to be used in specific biologically-inspired machine vision applications [40], [41]. There are 4 main advantages to using a DVS camera instead of a regular camera: 1) the input is frame-free: it consists of individual packets which hold an address (encoding the spatial position) and a timestamp. Therefore it is not necessary to process whole image scenes at a time, only events; 2) an event is only generated when something changes and so no time or resources are wasted processing visual information when nothing has actually happened; 3) the DVS 128 output is illumination independent as the triggering of a spike event is based purely upon pixel-level changes in the input. This is an extremely important issue for artificial vision systems as they need to be able to cope with the light levels in different environments; 4) minimal pre-processing is required as the camera directly encodes spike events which can be relayed straight into a spiking neural network. The DVS camera outputs raw events in AER (Address-Event Representation) format which consist of a 4-byte address and a 4-byte timestamp. The address encodes a spatial x, y position (in the range (0,127)(0,127)) for the event and also an event polarity of 1 or -1 signifying ON or OFF events respectively. Therefore the camera can register both when a pixel is activated and deactivated. In the current work the input motion sequences comprised of logged data from the camera: pre-recorded sequences of a bar-shaped object moving in one of eight directions (N, NE, E, SE, S, SW, W, NW) from which we extract and use only ON events. For more details on information extraction and processing of AER data see the jAER SourceForge wiki [42].

### Experimental Procedure and Analysis

For each experiment five randomly initialised networks were produced with afferent LGN and Cortical connectivity appropriate to the experiment. Ten different instances each of the eight input patterns (representing directions N, NE, E, SE, S, SW and NW) were presented to the network and the average of the firing rate in response to each direction was collected. Neuron preference and Selectivity Index (SI) were calculated using the vector average method as described in [10]. See Appendix for details. As is common in previous experimental and modelling studies we have visualised neuron direction preferences as a composite ‘map’: a 2D grid representation of the ‘cortex’ where pixels representing the spatial location of neurons are coloured according to their preferred direction. The Selectivity Index (SI) is a measure of the degree of selectivity of a neuron and takes values between 0.0 and 1.0 with 1.0 indicating exclusive preference for one direction or orientation.

## Results

### DS and OR is present in the initial architecture

**Figure 3:**
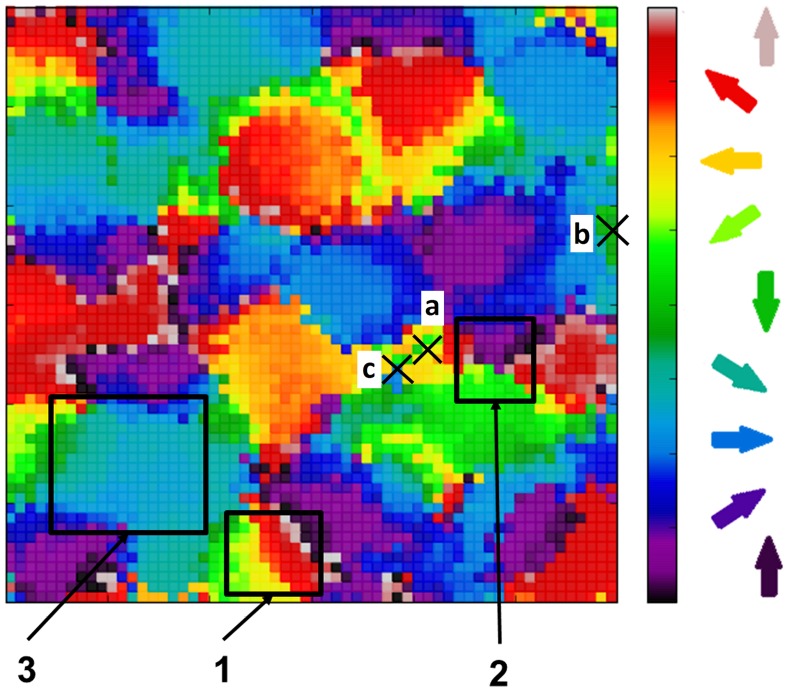
Direction Preference map present in default network architecture. The distribution of neuron preference for directional selectivity exhibits a similar patchy pattern to experimentally derived maps. In addition features seen in experimental maps such as regions of rapidly and smoothly changing preference (box 1), fracture lines with abrupt switch to opposite preference (box 2) and saddle points (box 3) are present. The letters a, b and c refer to neurons whose tuning curves are shown in Fig 4.

**Figure 4:**
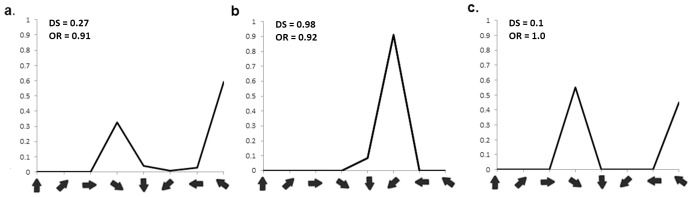
Tuning Curves for Selected Neurons in the default network. Tuning curves are plotted with normalised rate (ordinate) against direction (abscissa) and show cases where neurons have weak Directional Selectivity (a.), strong Directional Selectivity (b,) and Orientation Selectivity. The neurons' positions are indicated by letter on Fig 3.

Networks created with the architecture as shown in Figure 1 and as described in the Methods section exhibit distributed patchy activity on presentation of moving input generated by the DVS camera. When a plot was produced with Cortical neurons coloured according to their preferred direction (see Appendix for details of the calculation of neuron preference and selectivity) a distinct map reminiscent of an experimental DS map was generated. See Figure 3 for an example of the map from one network. This map exhibits some of the features which appear in maps derived from experimental data such as areas of rapidly and smoothly changing preference and fracture points where the preference changes abruptly by 180 degrees. For comparison see Fig 2 in [43] and Fig 5 in [44]. We also generated tuning curves for selected neurons. Figure 4 shows tuning curves for 3 neurons exhibiting weak DS (Fig 4a), strong DS (Fig 4b) and OR (Fig 4c). The location of the neurons are indicated by letter on Fig 3. Similar to the tuning curves shown in Fig 3c in [27], the OR selective neuron (our Fig 4c) occurs in a location where neuron preference changes rapidly and the weak and strong DS neurons (our Fig 4a and b) occur at locations where the preference changes more smoothly.

**Figure 5:**
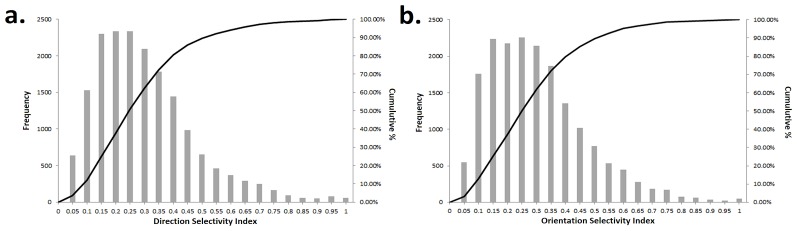
Distribution of Direction and Orientation selectivity strength. Here the Selectivity Index (SI) has been calculated for all neurons over all runs for both DS (a) and OR (b) and the data are presented as histograms with cumulative % graphs overlaid. The curves are very similar and also compare well to those found experimentally for pre-EO animals.

**Figure 6:**
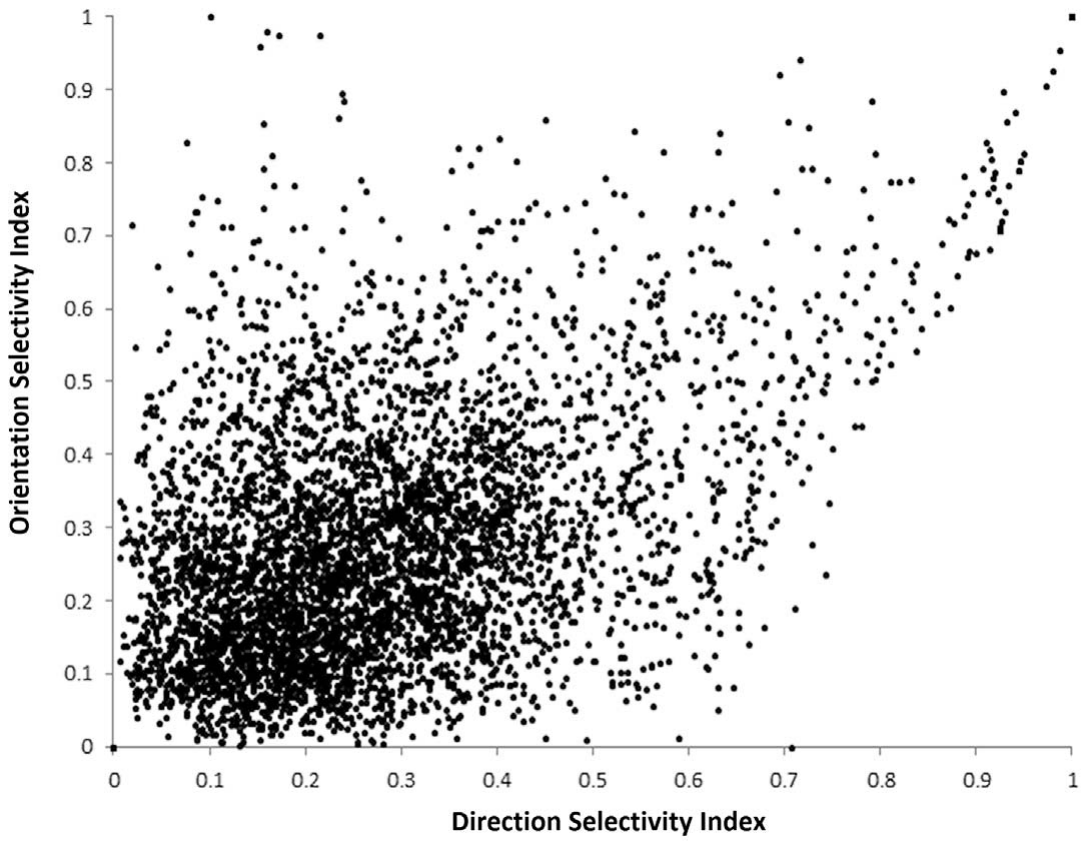
Comparison of individual neurons DS and OR strength. The scatter plot shows Orientation Selectivity Index (ordinate) against Direction Selectivity Index (abscissa) for all neurons over all runs. Various combinations are evident: high DS and OR, low DS/high OR and high DS/low OR.

We calculated the Selectivity Index (SI) for both DS and OR over all neurons and all runs and these results are presented as histogram/cumulative percentage plots in Fig 5. The distributions for both DS and OR are very similar to the experimental curves for the eyes closed condition shown in Fig 1b in [26]. Average DS and OR selectivity are both approximately 0.28. For OR this compares well with the value of ∼0.25 found at eye-opening in [26] but for DS the value is too high for an immature network (experimental value in [26] was ∼0.1). Fig 6 shows a scatter plot of OR vs DS for 5000 neurons randomly selected from all runs. We found that, as in biological networks, that there are actually a range of combinations of OR and DS: OR and DS can both be high, moderately high OR selectivity (>0.6) exists where DS is low (0.1–0.4). We also saw cases where DS is moderately high (0.6-0.8) and OR is low (0.1–0.4).

### The structure of LGN afferent receptive fields affects innate DS

**Figure 7:**
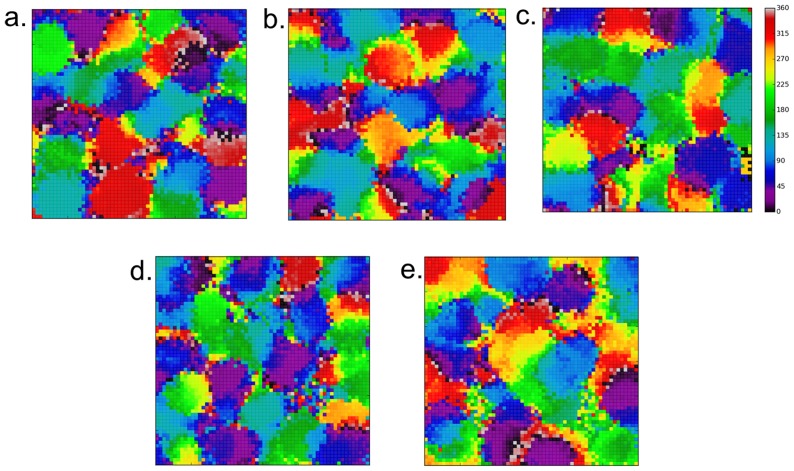
Direction Preference maps for different LGN afferent connectivity conditions. The figures in the top row show the effects of varying the LGN-Cortical connection field size on the arrangement of cortical responses. The cases are a) 3×3 connection field b) 5×5 connection field c) 7×7 connection field d) Random connectivity between the LGN and Cortical layers and e) LGN and Cortical layers fully connected. The characteristic distributed patches of activity in response to different patterns are present in all maps (although there are differences in the neuron selectivity see Table 3). Figures 7b (standard case CF 5×5; the same map as Fig 4) and 7c (CF 7×7) show best similarity to experimental maps, whilst 7d and 7e have much less smooth transitions of preference and bigger areas of uncertain preference (speckled patches).

In order to determine to what extent the afferent connectivity contributes to the innate DS we tested several different sizes of connection field: 3×3, 5×5 and 7×7. We also looked at extreme cases where connection fields were absent - where connectivity between the LGN and Cortical layer was random (with connection probability 0.2) and also full connectivity. Figure 7 shows plots of the composite maps for all scenarios. In all cases there was some kind of cortical ‘map’ with a distinctive patchy response. Figs 7b (standard case CF 5×5; the same map as Fig 3) and 7c (CF 7×7) show best similarity to experimental maps, whilst the others (and particularly 7d and 7e) have much less smooth transitions of preference and bigger areas of uncertain preference (speckled patches). Table 3 gives the Selectivity Index (SI) averaged over all neurons for all runs. We found that manipulating the form of the afferent connectivity affected neurons' selectivity but there was no clear trend of decreased selectivity with disruption to the afferent connectivity. Generally the differences in SI are modest, however, there is a distinct peak in selectivity for the 7×7 case. A Kruskal-Wallis test showed statistical significance in variation of SI across the different cases (p<0.05). We also performed post-hoc tests (pairwise comparison of all cases using the Mann-Whitney test) which showed that in all comparisons the difference in SI was significant.

**Table 3:** Direction Selectivity for Afferent Connectivity Experiments.

Afferent Connectivity	Selectivity Index
3×3 connection field	0.31
* **5×5 connection field**	**0.28**
7×7 connection field	0.36
Random	0.28
Full	0.31

*This is the standard case scenario.

To follow up the hint that the presence or absence of connection fields might be affecting the smoothness of the map we calculated direction preference gradient maps using the method described in [10] (also see Appendix for method). The gradient values give an indication of how smoothly the preference varies across the map and larger values indicate more abrupt jumps between preference. We calculated the average gradient for each map across 5 runs and compared between the different connectivity cases – see Table 4. We saw that there appeared to be a clear split between the cases where the connectivity was structured (3×3, 5×5, 7×7) and where it was not (Random. Full) with the latter having larger gradient values. A Kruskal-Wallis test showed overall significance (p<0.05) in the differences between all cases and post-hoc tests confirmed that this came entirely from the difference between the structured (3×3, 5×5, 7×7) and unstructured (Random, Full) groups whereas differences within these groups were not significant.

**Table 4:** Direction Preference Gradient for Afferent Connectivity Experiments.

Afferent Connectivity	Average Gradient
3×3 connection field	30.8
5×5 connection field	30.2
7×7 connection field	28.0
Random	37.4
Full	33.2

### Sufficient lateral inhibition is required for innate DS

**Figure 8:**
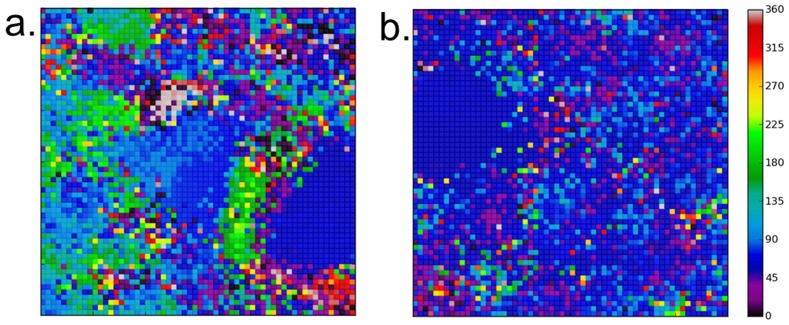
Direction Preference maps for reduced lateral inhibition. In both cases reducing lateral inhibition has almost completely disrupted the characteristic patchy structure seen in previous figures. When a 5×5 connection field is present (a) there are some patches of the same preference and topological arrangement of patches of similar preference but nowhere near as structured as the intact case. With full afferent connectivity (b) there is complete map disruption: most neurons are active for all of the motion directions and selectivity is very low (see Table 5).

We compared the cases for a 5×5 LGN connection field and full LGN-Cortical connectivity when the spatial range of lateral inhibition was drastically reduced. This was done by forcing the lateral inhibition to zero at distances greater than 8 units, effectively reducing the range from approximately 5× the excitatory range down to only 2x. Figure 8 shows plots of the composite maps for the two cases and indicates that in both cases the ‘map’ was significantly disrupted from the standard case. When a connection field was present (Fig 8a) we saw that there were some patches of the same preference and topological arrangement of patches of similar preference but nowhere near as structured as the map features shown in Figure 3. With full afferent connectivity (Fig 8b) the map was much more significantly disrupted. Table 5 shows statistics (to 2 decimal places) averaged over all neurons and five runs and complements what is seen in the plots. We found that the average neuron selectivity was very much lower than the cases where inhibitory connectivity was intact (see Table 3). The worst case was when there was both reduced lateral inhibition and full afferent connectivity. This is very apparent from Fig 8b. The difference between the two cases was found to be statistically significant (Mann-Whitney, p<0.05) indicating (as found in the previous experiment) that the form of the afferent connectivity contributes something to DS even though the effects of reduced lateral inhibition are dominant.

**Table 5:** Direction Selectivity for Lateral Inhibition Experiments.

Afferent Connectivity	Selectivity Index
Full	0.11
5×5 connection field	0.16

## Discussion

We have shown that with a simplified architecture and assumptions similar to previous modelling studies, DS is innate in the structure of our network and manifests on presentation of moving patterns without requiring a period of learning or ‘visual experience’. OR is also present in the network and the distribution of OR selectivity strength and average SI compares well with experimental findings for animals at eye-opening. The DS maps exhibit similar features to experimental maps although they are not identical. We believe that the differences might be due to the fact that we use real moving objects as stimuli. Experimental studies use moving sinusoidal gratings (as do most computational studies, for example [27]) which will ensure a consistent level of stimulation spatially and temporally. We also found that the average DS Selectivity Index is somewhat higher than that found in experiments, pre eye-opening – 0.28 compared to [26] where values of DS for animals after eye-opening at Postnatal Day (PND) <35 and >35 are approximately 0.15 and 0.25 respectively. We believe this may be due to the fact that our levels of lateral inhibition are set too high for an immature network.

As well as reproducing the findings of [27] with respect to DS and OR we have made a deeper study of the features of the network architecture that support innate DS by performing experiments that changed the afferent and lateral connectivity in various specific ways. We showed that the LGN layer afferent connection fields (which control the input available to cortical neurons) and the extent of lateral inhibition in the cortical layer interact to produce DS. Comparing the results of our experiments with varying afferent connectivity and lateral inhibition it is clear that the primary prerequisite for the presence of strong DS is sufficient lateral inhibition but maximal disruption to the DS was achieved when both afferent connection fields were absent and the radius of lateral inhibition was significantly reduced. In [27] the innate DS was attributed only to the cortical dynamics caused by inhomogeneities in the cortical connections and did not investigate the contribution from afferent connections. In terms of future work, there are aspects of our system that remain to be investigated which might impact on innate DS. Does the precise form of the spiking neuron model matter and do the values for membrane and synaptic time constants affect the response? We have used initial conditions in line with previous modelling works, but it would be interesting to establish if our proto-architecture is sensitive to specific initial distributions of connection weights and delays. We also believe it is important that such a proto-architecture is able to be tuned by visual experience as in real networks and future work will explore a learning scenario to see if experimental results can be reproduced. An example of such results is the work described in [26] which gives values for individual neuron selectivities before and after visual experience and also the global picture shown as a cumulative percentage graph which exhibits a distinct rightwards shift after visual experience showing that a large proportion of neurons increased their selectivity.

Our findings are potentially important for both future biological investigations and the creation of artificial systems. It seems reasonable that in natural systems a ‘proto-architecture’ might exist to canalize the development of something as vital as visual capability and ensure a degree of functionality early in the developmental process. It is equally important that this structure should be able to be tuned by visual experience when it becomes available. We believe that the concept of a proto-architecture delivering an initial capability which is modifiable by experience might be a general one with applications wider than that of just vision and should be a topic of investigation for developmental biologists. It is likely that new techniques or novel applications of existing techniques will be required to confirm or deny the presence of a proto-architecture and whether such a structure has a genetic basis. Complementary to our current work, more modelling studies are needed to establish that the form of cortical connectivity necessary for innate DS could arise from a genetic specification via a plausible developmental process. The recent modelling work of [45], [46] in growing cortical-like architectures using biologically plausible genetic/developmental processes indicates that this should be achievable.

The developmental approach to robotics has taken its inspiration from the fact that natural systems do not spring into being fully formed but undergo considerable periods of refinement and change, and in particular adapt in response to input from the environment. Crucial developmental processes in both pre- and early post natal life set the stage for later capability. The importance of such developmental processes, and in particular the gradual acquisition of capability, has been noted by previous robotics researchers exploring visual-motor coordination [47], [48]. However, these studies are still based on the concept of activity or ‘experience’ as the primary driver for development, often requiring significant training time to acquire a level of skill. We have shown that it is possible for a system for motion sensitivity to exist without the overhead of a learning mechanism and extensive training. For developmental robotics, this implies that a ‘tabular rasa’ may not be the most efficient starting point for artificial learning.

## Appendix

### Calculation of Neuron Preference and Selectivity

Having collected the average firing rate for each neuron in response to each direction, neuron orientation and direction preference was calculated using the vector average method described in [10]. For orientation, firing rates were averaged over the two opposite directions of motion as was done in [27] and the vector sum **V**(x,y) for each neuron was calculated using equations 9 and 10:
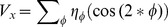
(9)

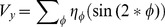
(10)


Where:


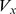
 and 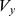 are the x and y component sums


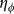
 is the firing rate for orientation 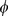


The preferred orientation 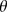 can then be found using equation 11:
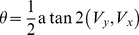
(11)


Note that equation 11 produces orientations in the range 0 to +/− 180 degrees. To convert to 0–180 range, 180 degrees need to be added to negative angles.

For direction preference the same method is used except that as direction is 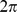-periodic, 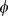 is not multiplied by 2 in equations 9 and 10 and there is no division by 2 in equation 11. Negative angles are converted to 0–360 range by adding 360 degrees.

The Selectivity Index (SI) is the magnitude of vector **V**. Normalised selectivity is calculated using equation 12.
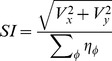
(12)


### Calculation of Preference Gradient

The direction preference gradient was calculated using the method described in [10]. Having calculated each neuron's direction preference, 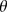, using equations (9–11), the differences between each neuron and its preceding neighbour in the x and y directions were computed:
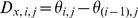
(13)

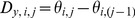
(14)


Where:


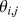
 is the direction preferred by neuron i,j.

The gradient magnitude is then calculated as:
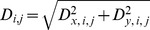
(15)

